# Socioeconomic Differentials in Employment Status and Involvement in Household Decision-Making Among Ever-Married Women in Nigeria

**DOI:** 10.3389/fsoc.2019.00049

**Published:** 2019-07-04

**Authors:** Rosemary O. Soetan, Mary O. Obiyan

**Affiliations:** ^1^Department of Economics, Obafemi Awolowo University, Ife, Nigeria; ^2^Department of Demography and Social Statistics, Obafemi Awolowo University, Ife, Nigeria

**Keywords:** women's empowerment, decision-making, employment status, education, Nigeria

## Abstract

Prior studies have assessed economic/instrumental dimensions of women's empowerment relative to its agency dimensions. This study assessed ever-married women's participation in the labor market as a form of agency for empowerment and household decision-making in Nigeria. The study utilizes secondary data from three national surveys of Nigeria's 2003, 2008, and 2013 Demographic and Health Surveys (DHS) to assess the differences and relationship between indices and the status of women's labor market participation and their empowerment or household decision-making over an 11-year period. Explanatory variables adopted were age group, religion, employment status, educational level, household wealth index, and region. Binomial logistic regression was used to predict the proxy variables of women's empowerment from the explanatory variables. Findings showed that women's access to paid employment, educational status, and the household wealth index improved their participation in household decision-making. However, the single factor that consistently increased the likelihood of an ever-married woman to be involved in all forms of household decision-making from 2008 to 2013 was household wealth. Also, religion affected the ability of ever-married women to participate in household decision-making. The ability of Muslim women to participate in all forms of decision-making decreased from 2008 to 2013. The study concluded that the factors that enhance household wealth will enhance married women's ability to be involved in household decision-making. Education of women is, however, a significant contribution to enhancing the balance of inequity between men and women in household decision-making. The attenuating effect of Islam on this prospect needs further investigation and interventions for married women living in Northern Nigeria.

## Introduction

Empowerment enables women to realize their potential and participate fully in every aspect of life (Heyzer, 2005; Mejiuni, 2013). It also improves their capacity for household decision- making with implications for their personal welfare and that of their children (Ruel et al., 2013). The need to empower women is a right in itself and a means of attaining economic growth (Eyben et al., 2008; Duflo, 2012), poverty reduction (Kabeer, 2003, 2005; Cornwall and Brock, 2005; Mejiuni, 2013), and good governance (McEwan, 2003). The inclusion of women's empowerment as part of the 2000–2015 Millennium Development Goals and the 2015–2030 Sustainable Development Goals shows the priority accorded.

While the concept of women's empowerment is a universal notion, its association with poverty reduction indicates that more attention needs to be paid to it in developing countries (Kishor and Subaiya, 2008; Ahmed et al., 2010). Institutions like the World Bank, United Nations agencies and local non-governmental organizations have implemented multiple programs aimed at improving the socioeconomic status of women. These programs create educational opportunities and access to micro-credit, among others (Malhotra and Mather, 1997; Kabeer, 2003; Duflo, 2012), with the aim of building financial independence capacity of women as a means for empowerment.

Empowerment increases the ability of women to participate in decision-making (Kabeer, 1999; Mejiuni, 2013). As women's access to wage employment improves, their contributions to the household and their negotiations improve, and their bargaining and eventual participation in household decision-making also improve (Schneebaum and Mader, 2013). Given the centrality of decision-making to the conceptualizations of power, decision-making therefore becomes the most frequently used measure of agency measure for empowerment (Malhotra and Schuler, 2005; Mejiuni, 2013).

The authors of this current study therefore examined the implications of women's labor market participation for their involvement in household decision-making in Nigeria, bearing in mind the predominant attention paid to the economic/instrumental dimensions of women's empowerment relative to its agency dimensions. This research examined the association between women's labor market participation and household decision-making in 2003, 2008, and 2013 and looked for consistencies in the pattern of association between women's labor market participation as well as participation in household decision-making over this period.

The study explored the direct and indirect route through which women's labor market participation was associated with participation in household decision-making.

This study is based on the extant micro-level phenomenon that women's access to education and employment opportunities result in a reduction in household poverty and increased women's empowerment (Brewster and Rindfuss, 2000; Engelhardt and Prskawetz, 2004; Nigeria National Planning Commission, 2007; Ahmed et al., 2010; Mejiuni, 2013). The authors acknowledge that choice and control are also indicators of women's empowerment (Malhotra and Schuler, 2005) and that there is a nexus between education and the empowerment of women (Mejiuni, 2013). The study focused on women's access to paid employment as a path for empowerment, measured by their participation in household decision-making.

## Method

The study area, Nigeria, is a middle-income country and the most populous in Africa, ranked seventh in the world with a total population of about 190 million people. The country is diversified in ethnicity and religion. It is divided into six geo-political zones, namely the South-West, South-South, South-East, North-East, North-West, and North-Central.

The data used for the study was extracted from the Nigerian Demographic and Health Survey (NDHS), a nationally representative survey. The NDHS is often conducted every 5 years, hence we extrapolated data from the three most recent surveys to show a change in outcomes over an 11-year period (2003–2013). There is currently no newer NDHS data. Although the 2018 NDHS survey has recently been concluded, the dataset is yet to be made available and accessible, hence this study analyzes NDHS 2003, 2008, and 2013.

The NDHS is a nationally representative survey that provides population and health indicators. The 2003 NDHS used two-stage cluster design sampling to select 365 clusters (200 in rural and 165 in urban areas) and chose 50 households systematically from each cluster. A total of 7,620 eligible respondents were successfully interviewed. The NDHS 2008 had a total of 888 clusters (286 urban and 602 rural) selected from a complete list of households, with an average of 41 households taken from each cluster through equal probability systematic sampling and a total 33,385 women were successfully interviewed. Lastly, NDHS 2013 used a three-stage stratified sampling design to select a total of 904 clusters (372 urban and 532 rural) with a fixed representative sample of 45 households per cluster and completed interviews of 38,948 respondents.

For the purpose of this study, only women aged 15–49 years who were “currently in union or living with a man” at the time of survey were included in the analysis. A total sample of *N* = 56,620 (which was comprised of *N* = 5,335 [NDHS 2003], *N* = 23,455 [NDHS 2008], and *N* = 27,830 [NDHS 2013]) was analyzed.

All variables used in this study were adopted from the women recode file. Some variables were measured as defined in the NDHS while others were recoded for the purpose of analysis. Explanatory variables such as “level of education,” “residence” and “household wealth index” were all adopted from the NDHS. The recoded variables were employment within past 12 months [none = 0, professional = 1, sales/services = 2, manual = 3], religion [Christianity = 1, Islam = 2, Traditional/Others = 3], and age group [15–24 = 1, 25–34 = 2, 35–44 = 3, 45+ = 4]. The independent variable was “Employment within the past 12 months” and the dependent variable “women empowerment” was proxied by three variables. They are (i) “decision on own healthcare,” (ii) “decisions on large household purchases,” and (iii) “decision on visits to her family or relatives.”

Each of the outcome variables is binary “0 and 1” and thus binomial logistic regression was adopted to assess the relationship between variables. Other covariates such as age group, religion, employment status, educational level, household wealth index, and region were controlled for in the analysis. Age group and religion were redefined and recoded. Age group was recoded into four groups: 15–24 years, 25–34 years, 35–44 years, and >45 years. Religion was recoded into Christian, Muslims, and others. Explanatory variables adopted were age group, religion, employment status, educational level, household wealth index, household headship, place of residence, and region. Age group and religion were redefined and recoded. Age group was recoded into four groups: 15–24 years, 25–34 years, 35–44 years, and >45 years.

Data analysis was conducted using STATA 15.0. Univariate analysis was conducted to show the frequency distribution of each respondent's characteristics. Bivariate analysis was conducted to highlight the associations between the dependent and independent variables using Pearson's chi-test with the level of significance set at *p* = 0.01. We used binomial logistic regression to predict the proxy dichotomous variables of women empowerment from the exposure variables. Appropriate weighting was applied throughout the analysis to ensure representativeness.

## Results

Table 1 shows the background characteristics of ever-married women in Nigeria by employment status in 2003, 2008, and 2013. There was a decrease in the proportion of ever- married women who were not employed who had these characteristics from 2003 through 2008 to 2013: those 35 years and older; those with primary and secondary level of education; those from the middle, richer, and richest household wealth quantiles; Christians; male head households; urban residents; and those residing in Northcentral, Northwest, Southeast and South-South Nigeria. Over the study period, unemployment increased among ever-married women from the poorest household wealth quantile (2003: 33.5; 2008: 38.3; 2013: 41.8%), with female household heads (2003: 15.4; 2008: 19.3, 2013: 19.4%), and from Southwest Nigeria.

**Table 1:** Women employment by individual socio-economic characteristics, NDHS 2003–2013.

**Individual variables**	**Employment categories of women**											
	**2003 (5,335)**				**2008 (23,455)**				**2013 (27,830)**			
	**None**	**Professional**	**Sales/**	**Agric/**	**None**	**Professional**	**Sales/**	**Agric/**	**None**	**Professional**	**Sales/**	**Agric/**
			**service**	**manual**			**service**	**manual**			**service**	**manual**
**AGE**												
15-24	51.3	2.2	28.7	17.7	46.9	1.4	26.1	25.5	49.0	1.5	30.1	19.4
25-34	31.8	5.2	40.0	23.0	27.5	5.4	39.5	27.6	27.8	5.3	45.0	21.9
35-44	21.9	8.8	46.3	23.0	19.9	7.1	43.9	29.1	18.0	7.2	51.9	22.9
45+	21.1	6.4	45.5	27.1	18.6	6.5	43.4	31.5	17.2	6.1	52.8	23.8
**EDUCATION**												
None	41.6	0.4	38.1	19.9	38.2	0.3	32.7	28.8	38.6	0.2	40.6	20.6
Primary	23.6	3.5	41.6	31.4	19.1	0.5	42.0	38.4	18.7	0.9	46.4	34.0
Secondary	26.4	9.8	43.5	20.3	22.8	5.7	47.9	23.6	21.2	5.0	54.0	19.8
Higher	16.1	57.3	18.8	7.9	20.2	48.3	26.6	4.9	19.7	47.4	28.1	4.8
**WEALTH**												
Poorest	33.5	0.4	29.5	36.5	38.3	0.1	26.7	34.9	41.8	0.1	37.4	20.8
Poorer	38.0	1.3	39.0	21.7	30.2	0.4	33.2	36.3	33.4	0.4	36.0	30.2
Middle	37.2	3.3	42.5	16.9	28.7	1.9	36.3	33.2	26.8	2.3	42.5	28.4
Richer	34.7	7.2	41.1	17.0	25.4	6.6	46.4	21.6	20.9	6.8	54.0	18.4
Richest	23.3	16.5	44.4	15.8	20.7	17.6	50.3	11.4	18.8	16.9	53.8	10.6
**RELIGION**												
Christians	18.6	10.6	33.4	37.5	18.5	9.4	37.8	34.3	16.3	10.0	45.5	28.3
Islam	17.9	9.9	45.6	26.7	37.8	1.6	38.7	21.9	37.3	2.0	43.7	17.0
Others	42.5	2.6	39.5	15.4	20.2	1.1	18.6	60.2	23.7	1.7	29.8	44.8
**HEADSHIP**												
Male	35.1	4.9	38.3	21.7	30.1	4.6	37.6	27.7	30.0	4.6	43.7	21.8
Female	15.4	11.8	48.2	24.6	19.3	9.4	41.2	30.1	19.4	9.9	49.1	21.6
**RESIDENCE**												
Urban	29.1	10.4	44.0	16.5	25.7	10.8	47.3	16.2	22.3	9.7	52.7	15.3
Rural	35.5	3.3	36.9	24.4	30.6	2.4	33.7	33.3	32.9	2.3	39.3	25.5
**REGIONS**												
N. Central	27.3	5.9	33.7	33.2	25.1	5.0	28.6	41.4	19.5	5.6	41.8	33.1
N. East	43.0	3.3	36.2	17.6	37.5	1.2	26.8	34.5	51.4	2.2	23.5	22.9
N. West	44.5	1.8	38.7	15.1	44.4	1.2	35.2	19.2	36.2	1.2	46.7	15.9
S. East	24.2	14.1	36.7	25.0	21.1	9.8	40.8	28.3	17.7	9.8	45.8	26.7
S. South	19.4	10.0	36.3	34.3	18.5	6.8	39.0	34.8	17.2	9.5	47.1	26.2
S. West	8.7	10.5	58.7	22.1	11.0	11.0	56.1	22.0	10.0	11.1	60.6	18.4

Table 2 shows the socioeconomic profile of ever-married women in Nigeria by their participation in different forms of decision-making—health, household purchase, and visit to family. More women in the 15–24 age group, with no education, with the poorest wealth index and not employed did not participate in any decision-making process in 2003, 2008, and 2013, respectively. It was only in South-West Nigeria that the proportion of women who did not engage in any of the decision-making processes decreased from 27.9% in 2003 to 16.4% in 2008 and 10.1% in 2013. Also, the proportion of women who did not engage in any of the decision-making process increased from 55.0% in 2003 to 57.3% in 2008 and 60.0% in 2013 in North-West Nigeria.

**Table 2:** Socio-economic characteristics of ever-married women by household decision-making in Nigeria, 2003–2013.

**Background characteristics**	**Participation in decision making**								
	**2003**			**2008**			**2013**		
	**Health**	**Household**	**Visit**	**Health**	**Household**	**Visit**	**Health**	**Household**	**Visit**
		**purchase**	**family**		**purchase**	**family**		**purchase**	**family**
	***p* < 0.001**	***p* < 0.001**	***p* < 0.001**	***p* < 0.001**	***p* < 0.001**	***p* < 0.001**	***p* < 0.001**	***p* < 0.001**	***p* < 0.001**
**AGE GROUP**									
15-24	12.9	8.8	26.8	31.4	26.7	43.6	25.8	23.9	35.6
25-34	22.2	18.5	38.6	45.5	39.5	56.8	40.4	39.2	48.7
35-44	29.2	27.5	43.1	49.4	43.1	60.2	45.9	45.2	53.9
45+	40.0	32.9	54.9	49.8	42.2	61.0	43.5	43.3	52.3
**EDUCATION**									
No Education	14.7	11.9	31.1	26.6	23.3	39.1	19.0	16.5	27.3
Primary Education	29.6	28.1	43.9	52.9	46.0	64.5	49.3	50.1	58.9
Secondary Education	33.2	26.2	45.1	61.3	51.9	70.9	59.4	59.6	68.3
Higher Education	50.0	39.8	63.4	70.2	60.1	78.8	70.7	69.1	77.8
**RELIGION**									
Christianity	35.9	29.9	48.5	64.1	56.7	74.4	64.8	66.1	73.4
Islam	43.8	41.6	52.1	27.7	22.8	39.7	22.3	19.5	30.9
Others	13.9	10.7	31.2	38.2	33.4	50.1	40.2	42.7	49.6
**HEADSHIP**									
Male	21.4	17.7	36.4	41.8	36.3	53.6	36.8	35.5	45.3
Female	44.1	41.4	58.3	62.2	52.4	69.0	59.8	59.9	69.9
**WEALTH**									
Poorest	18.7	15.6	36.0	25.7	23.3	37.4	15.5	13.3	24.7
Poorer	16.0	14.7	32.7	33.9	30.2	46.7	25.9	23.8	34.4
Middle	18.3	15.4	34.4	45.2	40.1	58.4	40.6	41.3	49.3
Richer	25.9	22.8	37.1	55.9	46.5	66.5	50.7	51.0	59.3
Richest	39.1	30.8	51.6	63.1	52.5	71.4	67.7	65.9	75.8
**RESIDENCE**									
Urban	30.9	24.9	44.5	54.6	45.3	63.7	54.3	52.9	63.1
Rural	19.8	17.2	35.3	38.8	34.3	51.1	29.9	28.9	38.3
**REGION**									
North-Central	23.2	12.6	23.9	54.5	54.2	67.4	42.1	44.2	46.8
North-East	10.5	9.8	41.5	26.7	21.9	37.8	26.3	19.4	36.6
North-West	10.9	9.5	26.7	18.9	17.3	29.9	16.2	15.0	23.5
South-East	59.3	49.6	75.3	58.6	53.0	66.0	63.3	61.9	73.2
South-South	36.2	39.3	43.2	64.4	53.2	77.7	65.7	71.0	73.3
South-West	51.3	39.2	58.6	69.0	53.9	80.4	72.4	70.9	85.9

**Figure 1:**
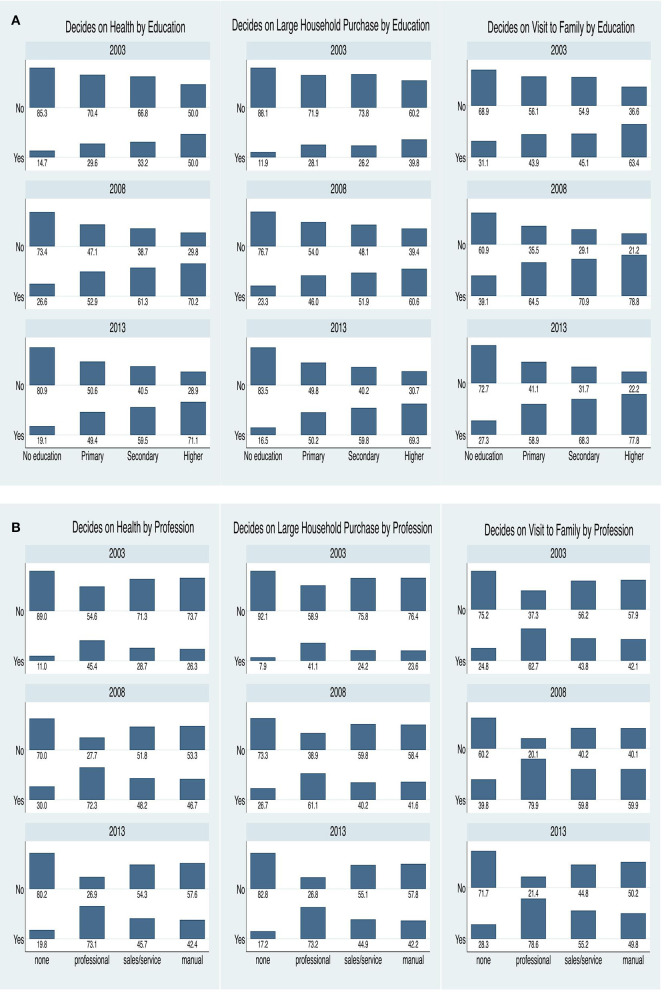
**(A)** Women's decision-making by education, 2003–2013. **(B)** Women's decision-making by profession, 2003–2013.

Figure 1 shows the proportion of ever-married women who could take decisions by education and employment status. Overall, a higher proportion of women with higher educational status could take decisions relating to health, major household purchase, and visit to family. Also, the proportion of ever-married women who could take decisions relating to health increased with the level of education. The proportion of women who could participate in decision-making regarding large household purchases increased over the years among women with employment status. Notably, the gap between women that had no employment and household decision-making widened increasingly over the years. The gap between those that had some level of education and those that had no education also widened and was most apparent when it came to decision-making on major household purchase.

Table 3 is a cross-tabulation of respondents' work status with involvement in household decision-making. Significantly more ever-married women (*p* < 0.001) with professional employment status were able to take part in decision-making regarding health, household purchases, and visits to family in years 2003, 2008, and 2013. For the same period, significantly less ever-married women (*p* < 0.001) were about to take part in household decision-making.

**Table 3:** Variations in household decision making by women's employment at ^*^95% C.I by year of survey.

**Variables**	**Coef**	**95% CI**	***p*-value**	**Coef**	**95% CI**	***p*-value**	**Coef**	**95% CI**	***p*-value**
**Profession**	**Health**			**Household purchase**			**Visit family**		
**HOUSEHOLD DECISION MAKING, 2003**									
None	0.12	(0.11 – 0.14)	p < 0.001	0.10	(0.08 – 0.11)	p < 0.001	0.27	(0.25 – 0.29)	p < 0.001
Professional	0.46	(0.40 – 0.52)		0.39	(0.34 – 0.45)		0.62	(0.57 – 0.68)	
Sales/Services	0.31	(0.29 – 0.33)		0.26	(0.24 – 0.28)		0.46	(0.44 – 0.48)	
Agric/Manual	0.31	(0.28 – 0.33)		0.27	(0.24 – 0.29)		0.46	(0.43 – 0.48)	
**HOUSEHOLD DECISION MAKING, 2008**									
None	0.29	(0.28 – 0.30)	p < 0.001	0.27	(0.26 – 0.28)	p < 0.001	0.40	(0.39 – 0.41)	p < 0.001
Professional	0.72	(0.69 – 0.74)		0.63	(0.60 – 0.65)		0.80	(0.77 – 0.82)	
Sales/Services	0.46	(0.44 – 0.47)		0.39	(0.38 – 0.40)		0.58	(0.57 – 0.59)	
Agric/Manual	0.47	(0.46 – 0.48		0.42	(0.41 – 0.43)		0.60	(0.59 – 0.61)	
**HOUSEHOLD DECISION MAKING, 2013**									
None	0.20	(0.19 – 0.21)	p < 0.001	0.18	(0.17 – 0.19)	p < 0.001	0.29	(0.28 – 0.30)	p < 0.001
Professional	0.72	(0.69 – 0.74)		0.73	(0.70 – 0.75)		0.77	(0.75 – 0.79)	
Sales/Services	0.46	(0.45 – 0.47)		0.45	(0.44 – 0.46)		0.56	(0.54 – 0.57)	
Agric/Manual	0.45	(0.44 – 0.46)		0.46	(0.45 – 0.47)		0.52	(0.51 – 0.54)	

* *the C.I at 95% is based on unweighted proportion*.

**Figure 2:**
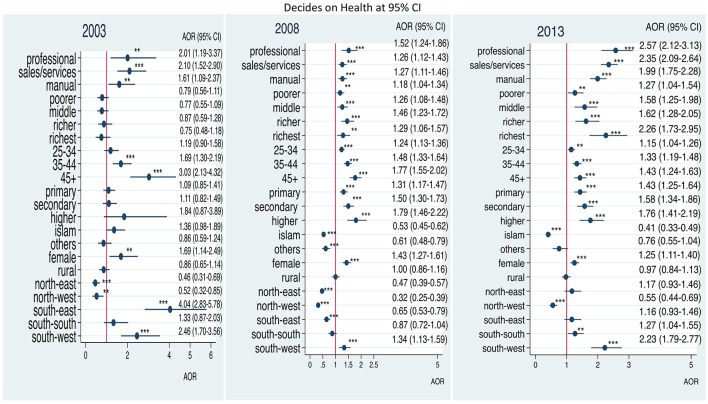
Adjusted odds ratios of decision-making on health by employment among ever-married women in Nigeria: 2003–2013. Adjusted factors: household wealth, age of respondent, level of education, religion, household headship, residence, geo-political zones. *p < 0.05; **p < 0.01; ***p < 0.001.

Figure 2 shows that the odds for women's participation in decision-making on health increased with education status when compared to those without education from 2003 to 2013. Also, women with higher household wealth status compared with women with the poorest household status, residents in rural areas compared with residents in urban area and residents in North-east Nigeria compared with residents in North-central Nigeria had better participation in decision-making regarding health from 2003 to 2013. The odds decreased for Muslim women when compared with Christian women and for female-headed households over the same period. The difference within the age groups also decreased over the same period.

**Figure 3:**
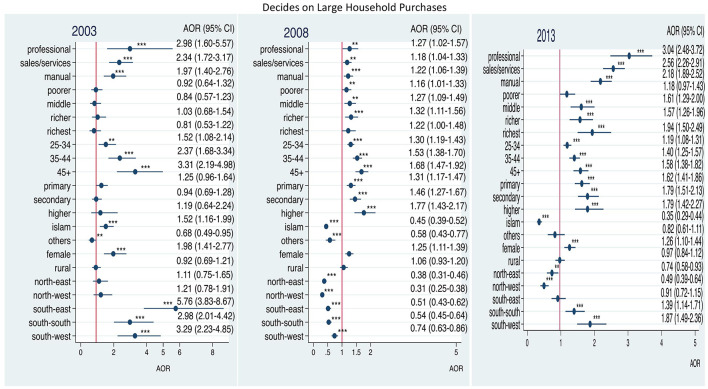
Adjusted odds ratios of decision-making on large household purchases by employment among ever-married women in Nigeria: 2003–2013. Adjusted factors: household wealth, age of respondent, level of education, religion, household headship, residence, geo-political zones. *p < 0.05; **p < 0.01; ***p < 0.001.

Figure 3 shows that the odds for women's participation in decision-making on major household purchases increased with education status when compared with women without education and with higher household wealth status compared with women with the poorest household status from 2003 to 2013. The odds decreased for women who were resident in Northeast and Northwest Nigeria over the same period. The odds also decreased for women who were resident in all regions compared with Northcentral Nigeria; and for gainfully employed women when compared with those who were not employed from 2003 to 2008.

**Figure 4:**
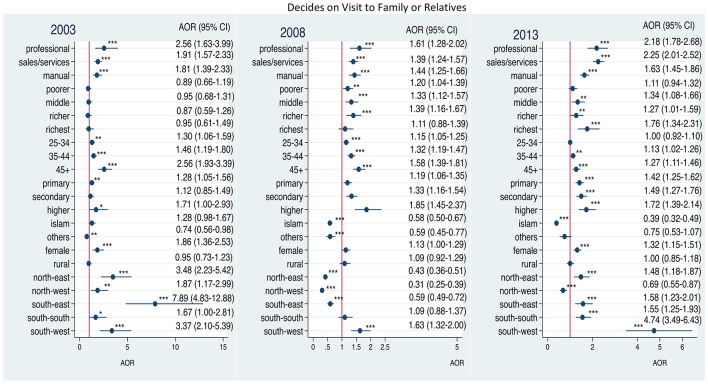
Adjusted odds ratios of decision-making on visit to family or relatives by employment among ever-married women in Nigeria: 2003–2013. Adjusted factors: household wealth, age of respondent, level of education, religion, household headship, residence, geo-political zones. *p < 0.05; **p < 0.01; ***p < 0.001.

Figure 4 shows that the odds for women's participation in decision-making regarding visits to family increased with household wealth status compared with women with the poorest household status from 2003 to 2013. The odds decreased over the same period for women who were resident in Northwest Nigeria and for Muslim women.

## Discussion

The results of the study highlight the dynamics of household decision-making in Nigeria over an 11-year period. The study showed that that women's access to paid employment, educational status, and household wealth index improved their participation in household decision-making. However, the single factor that consistently increased the likelihood of an ever-married woman to be involved in all forms of household decision-making from 2008 to 2013 was household wealth. The study also found that religion affected the ability of ever-married women to participate in household decision-making. The ability of Muslim women to participate in all forms of decision-making decreased from 2008 to 2013.

What the authors seemed to observe is a phenomenon where the balance of power in household decision-making improved as the household wealth index improved. This balance of power also seemed to improve as the educational and employment status of women improved. A recent study in Spain shows that when demographics, family, and labor market characteristics were controlled for, the educational status of both men and women were the major influencers of an egalitarian household decision-making process (Albert and Escardíbul, 2017). In part, the findings of Albert and Escardíbul's study in Spain is consistent with the findings of this current study and with Mejiuni's study in Nigeria which reported that educational status influenced the household decision-making process for women (Mejiuni, 2013).

Other sociocultural factors, such as religion and geographical factors like residential location, also influenced household decision-making for women. This study shows that the Islamic religion reduced the decision-making ability of ever-married women over the study period. Low decision-making power is pertinent among women who reside in Northern Nigeria where Islam is dominantly practiced. These women were reported to be less able to take part in household decision-making compared to women in Southern Nigeria where other religion(s) are dominant. Mercy (2017) affirmed this finding in her study that, more than any other geographical settings in Nigeria, women in northern Nigeria have very limited educational opportunities and very few of their children who were girls completed their primary education, mostly due to early marriage, poverty, and oftentimes religious reasons.

The ability of women to take part in household decision-making reflects their autonomy. Largely, this study suggests that the autonomy of married women may have improved over the last 11 years in Nigeria. Although the educational disparity between males and females persists (British Council, 2014) and is worse at the tertiary level of education, despite the increase in school enrolment figures over the years (Onwuameze, 2013), the findings of this current study show that educational enrolment for women has improved over the study period. Other studies have shown that women who are educated and especially those who have secondary or more education will likely have more autonomy to make unilateral or joint decisions regarding their health and other family members (Ejembi et al., 2004; Mejiuni, 2013).

Consistent with other studies (Amugsi et al., 2016), the findings of this current study have implications for maternal and child health because improved autonomy of women would result in improved household decision-making regarding major household purchases and improved nutritional status of members of the household. For a country like Nigeria where there has been no substantial improvement in the under-5 mortality index, improving the ability of women to take decisions on health and improving her social capital through her ability to take decisions to visit family members can increase maternal health service utilization (Grown et al., 2005; Ahmed et al., 2010; Obiyan and Kumar, 2015) and maternal under-5 mortality (Fantahun et al., 2007; Varkey et al., 2010).

Summarily, the authors of this current study noticed that women's levels of participation in household decision-making varied significantly across the six geopolitical zones in Nigeria. Women residing in geopolitical zones located in northern Nigeria had lower household decision-making autonomy when compared with women in Southern geopolitical zones. Some studies attributed this to lower level of education (UNICEF, 2007; Mejiuni, 2013; Mercy, 2017) and lower employment status (Bano, 2018). This has further implications for health conditions and welfare status, given the report that maternal mortality and under-5 mortality rates are worse in Northern Nigeria (Doctor et al., 2011; Wollum et al., 2015).

## Conclusion

This study showed that over the last 11 years, there have been changes in the status of women in ways that affect their decision-making power. Education of women has proved to be a significant contribution to enhancing a balance in equity between men and women in household decision-making. The attenuating effect of Islam on this prospect needs further investigation and interventions for married women living in Northern Nigeria. With respect to variation on the influence of Islamic religion on women decision-making between the North and South, we suggest that adherence to stricter practices of Islam in the North relative to the South could have accounted for the variation. Such adherence to religious practice increases male dominance and male dependency. Islamic extremism has also increased over the years. It could also be partly a function of the relatively higher level of exposure to western education by Muslim women in the South. However, this opens a frontier for further research.

Like all cross-sectional studies, what this study has been able to establish is an association between variables rather than a cause-effect relationship. The findings can, at best, help establish a hypothesis for a study that determines a cause-effect relationship between household decision-making and employment status of ever-married women in Nigeria.

## Ethics Statement

The study employed a secondary dataset of the Nigerian Demography and Health Surveys. This is a nationally representative survey conducted by the Measure DHS/ICF International in partnership with Nigerian National Population Commission. All protocols and survey instrument were approved by the Institutional Review Board (IRB) of Nigeria and IRB of the ICF International. The corresponding author obtained approval from Measure DHS to access, use and analyze the dataset of the study area.

## Author Contributions

RS conceptualized the study. MO developed the data analysis plan, conducted the data analysis, and developed the framework for the manuscript. RS and MO contributed to the development of the manuscript, reviewed the final paper and gave consent to its publication.

### Conflict of Interest Statement

The authors declare that the research was conducted in the absence of any commercial or financial relationships that could be construed as a potential conflict of interest.

## References

[B1] Ahmed S. Creanga A. A. Gillespie D. G. Tsui A. O. (2010). Economic status, education and empowerment: implications for maternal health service utilization in developing countries. PLoS ONE 5:e11190. 10.1371/journal.pone.0011190 20585646 PMC2890410

[B2] Albert C. Escardíbul J. O. (2017). Education and the empowerment of women in household decision-making in Spain. Int. J. Consum. Stud. 41, 158–166. 10.1111/ijcs.12326

[B3] Amugsi D. A. Lartey A. Kimani-Murage E. Mberu B. U. (2016). Women's participation in household decision-making and higher dietary diversity: findings from nationally representative data from Ghana. J Health Popul Nutr. 35:16. 10.1186/s41043-016-0053-1 27245827 PMC5026004

[B4] Bano M. (2018). Religion and female empowerment: evidence from Pakistan and Northern Nigeria. Can J Dev Studies/Revue Can d'études du Dév. 40, 1–19. 10.1080/02255189.2018.1470967

[B5] Brewster K. L. Rindfuss R. R. (2000). Fertility and women's empowerment in industrialized nations. Annu. Rev. Sociol. 26, 271–296. 10.1146/annurev.soc.26.1.271

[B6] British Council. (2014). Girls' education in Nigeria. Report 2014: Issues, Influencers and Actions. Retrieved from https://www.britishcouncil.org/sites/default/files/british-council-girls-education-nigeria-report.pdf (accessed August 27, 2018).

[B7] Cornwall A. Brock K. (2005). What do buzzwords do for development policy? A critical look at “participation,” “empowerment” and “poverty reduction.” Third World Q. 26, 1043–1060. 10.1080/01436590500235603

[B8] Doctor H. V. Bairagi R. Findley S. E. Helleringer S. Dahiru T. (2011). Northern nigeria maternal, newborn and child health programme: selected analyses from population-based baseline survey. Open Demograph. J. 4, 11–21. 10.2174/1874918601104010011

[B9] Duflo E. (2012). Women empowerment and economic development. J. Econ. Lit. 50, 1051–1079. 10.1257/jel.50.4.1051

[B10] Ejembi C. Alti-Muazu M. Chirdan O. Ezeh H. Sheidu S. Dahiru T. (2004). Utilization of maternal health services by rural hausa women in zaria environs, northern nigeria: has primary health care made a difference? J. Comm. Med. Primary Health Care 16, 47–54. 10.4314/jcmphc.v16i2.32414

[B11] Engelhardt H. Prskawetz A. (2004). On the changing correlation between fertility and female employment over space and time. Eur. J. Population/Revue européenne de Démograph. 20, 35–62. 10.1023/B:EUJP.0000014543.95571.3b

[B12] Eyben R. Kabeer N. Cornwall A. (2008). Conceptualising Empowerment and the Implications for Pro-Poor Growth: a Paper for the DAC Poverty Network. Brighton: Institute of Development Studies.

[B13] Fantahun M. Berhane Y. Wall S. Byass P. Högberg U. (2007). Women's involvement in household decision-making and strengthening social capital—crucial factors for child survival in ethiopia. Acta Paediatr. 96, 582–589. 10.1111/j.1651-2227.2007.00147.x 17306012 PMC2049066

[B14] Grown C. Gupta G. R. Pande R. (2005). Taking action to improve women's health through gender equality and women's empowerment. Lancet 365, 541–543. 10.1016/S0140-6736(05)17872-6 15705464

[B15] Heyzer N. (2005). Making the links: women's rights and empowerment are key to achieving the millennium development goals 1. Gender Dev. 13, 9–12. 10.1080/13552070512331332272

[B16] Kabeer N. (1999). Resources, agency, achievements: reflections on the measurement of women's empowerment. Dev. Change 30, 435–464. 10.1111/1467-7660.00125

[B17] Kabeer N. (2003). Gender Mainstreaming in Poverty Eradication and the Millennium Development Goals: A handbook for Policy-Makers and other Stakeholders. London: Commonwealth Secretariat. 10.14217/9781848598133-en

[B18] Kabeer N. (2005). Gender equality and women's empowerment: a critical analysis of the third millennium development goal 1. Gender Dev. 13, 13–24. 10.1080/13552070512331332273

[B19] Kishor S. Subaiya L. (2008). Understanding Womens Empowerment: A Comparative Analysis of Demographic and Health Surveys (DHS) Data. DHS Comparative Reports 20. Calverton, MD: Macro International.

[B20] Malhotra A. Mather M. (1997). Do schooling and work empower women in developing countries? Gender and domestic decisions in Sri Lanka. Sociol. Forum. 12, 599–630. 10.1023/A:1022126824127

[B21] Malhotra A. Schuler S. R. (2005). Women's Empowerment as a variable in International Development. in Measuring Empowerment: Cross-disciplinary Perspectives, ed Narayan D. (Washington DC: The World Bank), 71–88.

[B22] McEwan C. (2003). “Bringing government to the people”: Women, local governance and community participation in South Africa. Geoforum 34, 469–481. 10.1016/S0016-7185(03)00050-2

[B23] Mejiuni O. (2013). Women and Power: Education, Religion and Identity. Dakar: Council for the Development of Social Science Research in Africa (CODESRIA). Available at: https://www.codesria.org/spip.php?article1777&lang==eng (accessed April 7, 2019).

[B24] Mercy O. (2017). An assessment of factors militating against girl child education in Nigeria. Int. J. Adv. Multidiscipl. Soc. Sci. 3, 49–54. 10.5923/j.jamss.20170302.03

[B25] Nigeria National Planning Commission. (2007). National Economic Empowerment and Development Strategy-2: NEEDS-2. Abuja: National Planning Commission.

[B26] Obiyan M. O. Kumar A. (2015). Socioeconomic inequalities in the use of maternal health care services in Nigeria: trends between 1990 and 2008. Sage Open 5:2158244015614070. 10.1177/2158244015614070

[B27] Onwuameze N. C. (2013). Educational opportunity and inequality in nigeria: assessing social background, gender and regional effects. (PhD, thesis), University of Iowa.

[B28] Ruel M. T. Alderman H. Maternal Child Nutrition Study Group. (2013). Nutrition-sensitive interventions and programmes: how can they help to accelerate progress in improving maternal and child nutrition? Lancet 382, 536–551. 10.1016/S0140-6736(13)60843-0 23746780

[B29] Schneebaum A. Mader K. (2013). The Gendered Nature of Intra-Household Decision Making in and across Europe. Department of Economics Working Papers, wuwp157, Vienna: Vienna University of Economics and Business.

[B30] UNICEF. (2007). Information Sheet on Girls Education Project. Abuja: Nigeria Country Office. United Nations Children Education Fund.

[B31] Varkey P. Mbbs, Kureshi S. Lesnick T. (2010). Empowerment of women and its association with the health of the community. J. Womens Health 19, 71–76. 10.1089/jwh.2009.1444 20088661

[B32] Wollum A. Burstein R. Fullman N. Dwyer-Lindgren L. Gakidou E. (2015). Benchmarking health system performance across states in nigeria: a systematic analysis of levels and trends in key maternal and child health interventions and outcomes, 2000–2013. BMC Med. 13:208. 10.1186/s12916-015-0438-9 26329607 PMC4557921

